# Altered GH-IGF-1 Axis in Severe Obese Subjects is Reversed after Bariatric Surgery-Induced Weight Loss and Related with Low-Grade Chronic Inflammation

**DOI:** 10.3390/jcm9082614

**Published:** 2020-08-12

**Authors:** Paula Juiz-Valiña, Lara Pena-Bello, Maria Cordido, Elena Outeiriño-Blanco, Sonia Pértega, Barbara Varela-Rodriguez, Maria Jesus Garcia-Brao, Enrique Mena, Susana Sangiao-Alvarellos, Fernando Cordido

**Affiliations:** 1 Endocrine, Nutritional and Metabolic Diseases Group, Faculty of Health Sciences, University of A Coruña, 15001 A Coruña, Spain; Paula.Juiz.Valina@sergas.es (P.J.-V.); Maria.Lara.Pena.Bello@sergas.es (L.P.-B.); Maria.Cordido.Carro@sergas.es (M.C.); barbara.varela.rodriguez@gmail.com (B.V.-R.); 2 Instituto de Investigación Biomedica (INIBIC), University Hospital A Coruña, 15001 A Coruña, Spain; 3 Centro de Investigaciones Científicas Avanzadas (CICA), Campus de San Vicente de Elviña, 15008 A Coruña, Spain; 4 Department of Endocrinology, University Hospital A Coruña, 15001 A Coruña, Spain; Elena.Outeirino.Blanco@sergas.es; 5 Clinical Epidemiology and Biostatistics Unit, University Hospital A Coruña, 15001 A Coruña, Spain; Sonia.Pertega.Diaz@sergas.es (S.P.); Enrique.Mena.del.Rio@sergas.es (E.M.); 6 Department of Digestive and General Surgery, University Hospital A Coruña, 15001 A Coruña, Spain; MA.Jesus.Garcia.Brao@sergas.es

**Keywords:** obesity, bariatric surgery, endocrine disorders, somatotropic axis, GH, IGF-1

## Abstract

Endocrine disorders are common in obesity, including altered somatotropic axis. Obesity is characterized by reduced growth hormone (GH) secretion, although the insulin-like growth factor-1 (IGF-1) values are controversial. The aim of this study was to evaluate the effect of weight loss after bariatric surgery in the GH–IGF-1 axis in extreme obesity, in order to investigate IGF-1 values and the mechanism responsible for the alteration of the GH–IGF-1 axis in obesity. We performed an interventional trial in morbidly obese patients who underwent bariatric surgery. We included 116 patients (97 women) and 41 controls (30 women). The primary endpoint was circulating GH and IGF-1 values. Circulating IGF-1 values were lower in the obese patients than in the controls. Circulating GH and IGF-1 values increased significantly over time after surgery. Post-surgery changes in IGF-1 and GH values were significantly negatively correlated with changes in C-reactive protein (CRP) and free T4 values. After adjusting for preoperative body mass index (BMI), free T4 and CRP in a multivariate model, only CRP was independently associated with IGF-1 values in the follow-up. In summary, severe obesity is characterized by a functional hyposomatotropism at central and peripheral level that is progressively reversible with weight loss, and low-grade chronic inflammation could be the principal mediator.

## 1. Introduction

Obesity is an emerging condition. The prevalence of obesity has experienced a continuous increase in most countries since 1980. The disease burden related to high body mass index (BMI) has progressively increased since 1990 mainly due to cardiovascular disease [1]. The prevalence of obesity in Spain in 2010 was 22.9% (24.4% among men and 21.4% among women) [2]. Furthermore, it is estimated that the prevalence of obesity and severe obesity will continue to increase [3]. A weight loss of 5% improves metabolic function in numerous organs concurrently, and progressive weight loss causes dose-dependent alterations in key adipose tissue biological pathways [4,5]. Bariatric surgery compared with non-surgical obesity management, has been shown to produce more marked improvements in comorbidities associated with obesity and a higher decrease in all-cause mortality [6].

Excess body fat is associated with endocrine alterations, including thyroid dysfunction, central resistance to thyroid hormones [7,8] and decreased growth hormone (GH) secretion [9,10,11]. The altered GH secretion of obesity can be reversed by body weight normalization [12]. The most remarkable secretory capacity appeared when obese subjects were treated with GH-releasing hormone plus GH-Releasing Peptide-6, which gave rise to a substantial GH response [13]. In spite of the marked hyposomatotropism circulating insulin-like growth factor-1 (IGF-1) levels are conflicting in obesity. Circulating IGF-1 levels have been described to be normal [14,15,16,17,18], decreased [19,20] or even increased [21,22,23] in obese patients. Moreover, peak stimulated GH and IGF-1 reveal significant discrepancies in identification of obese subjects with reduced GH secretion [24]. The GH peak, compared with IGF-1, has broader connections with different parameters of endogenous GH secretion and peak stimulated GH and IGF-1 demonstrate significant discordance in the diagnosis of reduced GH secretion in obese patients [24]. The regulation of IGF-1 is complex and many factors in addition to GH have a role, such as hypothalamic neuropeptides, ghrelin, insulin, circulating free fatty acids, nutritional status and IGF-1 binding proteins (IGFBPs) [25,26]. Population studies in adults show that IGF-1 secretion is dependent on BMI with an inverse U-shaped curve and higher levels between a BMI kg/m^2^ of 30–35, and decreased values in more severe obese and underweight subjects [27]. Additionally, there are multiple and complex connections between circulating IGF-1 values and several health conditions and diseases [28]. The complex nature of disease- and subgroup-specific associations, together with methodological factors can be considered responsible for conflicting findings in previous works [28]. Even though the GH–IGF-1 axis has been evaluated before and after weight loss, most studies were performed on small groups of patients, did not include a control group or evaluated the variation of GH–IGF-1 over a limited time after the intervention and the results are inconsistent [12,19,29,30].

The aim of this study was to evaluate the effect of weight-loss evolution over time after bariatric surgery in the GH–IGF-1 axis in patients with extreme obesity, in order to investigate the mechanism responsible for the alteration of the GH–IGF-1 axis in extreme obesity. We hypothesized that the GH–IGF-1 axis is altered during morbid obesity due to hormonal and inflammatory adjustments, and that weight loss induced by bariatric surgery increases GH and IGF-1 values.

## 2. Patients and Methods

### 2.1. Patients and Controls

We performed an interventional trial evaluating patients with severe obesity who underwent bariatric surgery at the University Hospital of A Coruña, between January 2016 and December 2019. We included in our study 116 patients (97 women) and 41 controls (30 women) of similar age and sex selected from a pool of volunteers available to our unit. This sample size allows the estimation of change in GH and IGF-1 values after bariatric surgery with a 95% confidence and a precision of ±0.6 µg/L and ±20.5 µg/L, respectively. For this sample size estimation, standard deviation values obtained from a previous study was used [8]. The inclusion criteria for bariatric surgery were to be between 18 and 65 years old, have a BMI > 40 kg/m^2^ (or >35 kg/m^2^ and at least one serious obesity-related health problem, such as diabetes, high blood pressure or sleep apnea), failure of previous nonsurgical attempts at weight reduction, expectation that patient will adhere to postoperative care and follow-up visits with team members. Exclusion criteria of the obese patients were current drug or alcohol abuse, uncontrolled severe psychiatric illness, reversible endocrine or other disorders that can cause obesity, lack of comprehension of benefits, risks, alternatives, expected outcomes, and lifestyle changes required with bariatric surgery. A multidisciplinary team that includes a bariatric surgeon, an endocrinologist, and a psychiatrist evaluate all patients considered for bariatric surgery and based on the clinical characteristics (BMI, age, health problems) allocate the patient to sleeve gastrectomy or Roux-en-Y gastric bypass (RYGB). Exclusion criteria of the controls were the presence of diabetes mellitus or other medical problems, none of the controls were taking any drugs. The study protocol was approved by our center’s ethics committee (Xunta de Galicia), approval code number: 2014/135, and written informed consent was obtained from all patients and controls. All of the studies were conducted in accordance with the Declaration of Helsinki. 

### 2.2. Parameters Analyzed

The following data were analyzed: age, sex, BMI, body fat percentage, excessive BMI loss in percentage, GH, IGF-1, TSH, free T4 (FT4), insulin, HOMA-IR, C-reactive protein (CRP), C peptide, and the type of bariatric surgery performed (Roux-en-Y gastric bypass (RYGB) or sleeve gastrectomy (SG)). The data were assessed once in the controls, and before and one, three, six and twelve months after bariatric surgery in the obese patients. All blood samples were collected after an overnight fast in the morning between 8:00 a.m. and 9:00 a.m. and immediately centrifuged, separated and frozen at −80 °C. The primary end point was circulating IGF-1 and GH. The secondary end point was the influence of the thyroid axis and inflammation on circulating IGF-1.

### 2.3. Analytical Procedures

Serum GH (μg/L) and IGF-1 (ng/mL) were determined by a chemiluminescent assay (Immulite, EURO/DPC, Llanberis, UK) as previously published [7]. Serum TSH (mIU/L) and FT4 (ng/dL) levels were determined on serum obtained from blood samples, by chemiluminescent immunoassay (ADVIA Centaur, Siemens, Deerfield, USA) as previously published [7]. Serum Insulin (µU/mL) was determined with a chemiluminescent immunometric assay (Immulite 2000 Insulin, DPC, Los Angeles, CA, USA) as previously published [7]. Serum wide range C-reactive protein was determined with a latex-enhanced immunoturbidimetric assay (ADVIA Chemistry Wide Range CRP, Siemens Healthcare, Erlangen, Germany). Glucose (mg/dL) was measured with an automatic glucose oxidase method (Roche Diagnostics, Mannheim, Germany). All samples from a given subject were analyzed in the same assay run. Total fat mass and fat-free mass percentage was calculated through bioelectrical impedance analysis (BIA). The BIA measurements were taken using a tetrapolar bioimpedantiometer BC-418 Segmental Body Composition Analyzer (TANITA). The participants were examined while lightly dressed, and barefoot, placing the feet on the metal footprints, grasping the hand grips with both hands, without moving and in a standing position. The measurement process was standard and was strictly supervised.

### 2.4. Calculations

Excessive BMI loss in percentage was calculated using the formula: ((preoperative BMI-current BMI)/(preoperative BMI-25)) × 100.

Insulin sensitivity (IS) was measured with HOMA-IR with the formula: fasting serum insulin (µU/mL) × fasting plasma glucose (mmol/L)/22.5.

### 2.5. Statistical Analysis

Descriptive analysis was used to determine the baseline characteristics of both the patients and the controls. Continuous data are expressed as median and interquartile range (IR). Non-numerical variables are expressed as frequencies and percentages.

The control subjects and obese patients were compared using the Mann–Whitney test for quantitative parameters and the Chi-squared and Fisher’s exact tests for qualitative parameters. The Wilcoxon signed-rank test was used to compare the preoperative and post-surgical values in the obese patients, since the difference of pairs in the variables studied did not follow a normal distribution. Normality assumption was tested by means of the Kolmogorov–Smirnov test.

To assess the overall association of IGF-1 and GH with anthropometric, biochemical and hormonal data during the post-surgery period, repeated measures correlation was calculated [31].

Generalized estimating equations (GEE) models, with the autoregressive correlation structure, were used to evaluate the trajectory of postoperative IGF-1 and GH, as well as to determine factors associated with their changes after bariatric surgery. Bivariate GEE models were used in order to observe the effect of each variable on the change in IGF-1 and GH, taking time into account. Those features significantly associated with IGF-1 and GH values in the bivariate analysis were finally included in a multivariate GEE model.

For the statistical analyses, SPSS v 24.0, and R v 3.5.1 (with the geepack and rmcorr packages added) were used. Bilateral *p*-values < 0.05 were considered as statistically significant.

## 3. Results

### 3.1. Characteristics of the Obese Patients and the Control Group

The type of surgery and anthropometric characteristics of the obese patients before surgery (*n* = 116) and the control subjects (*n* = 41) are presented in Table 1. Most patients were women (*n* = 97) (Table 1). Before surgery, 28.5% of the patients had diabetes (10.4% insulin treated and 18.1% non-insulin treated), 23.3% dyslipidemia, and 34.5% hypertension. Twelve months after bariatric surgery, 9.1% of the patients had diabetes (3% insulin treated and 6.1% non-insulin treated), 6% dyslipidemia, and 12.9% hypertension. All patients underwent RYGB or SG. Among the 41 controls studied, 30 were women (Table 1). The two groups had similar sex and age as designed by the matching criteria.

### 3.2. Fasting Serum Levels

Fasting glucose and hormones are presented in Table 2. IGF-1 levels were lower in the obese group than in than in normal-weight healthy volunteers; 79.5 (56.6; 95.0) vs. 138.0 (111; 174) for the obese and control group, respectively. HOMA-IR levels were higher in the obese group than in normal-weight healthy volunteers; 1.4 (0.6; 2.7) vs. 0.8 (0.6; 1.5) for the obese and control group, respectively.

### 3.3. Time Course of the Clinical and Analytical Parameters

The median excessive BMI loss in percentage (EBMIL) after bariatric surgery was 36.4%, 48.2%, 61.8% and 73.8% at one, three, six and twelve months, respectively.

The biochemical and hormonal parameters in obese patients (*n* = 116) before and twelve months after bariatric surgery are presented in Table 3. IGF-1 significantly increased in the obese patients after weight loss induced by bariatric surgery; 79.5 (56.6; 95.0) vs. 110.5 (88.0; 129.5) for the obese patients before and twelve months after surgery, respectively. GH levels significantly increased in the obese patients after bariatric surgery weight loss; 0.3 (0.1; 0.8) vs. 2.1 (0.3; 4.8) for the obese patients before and twelve months after bariatric surgery, respectively.

**Figure 1:**
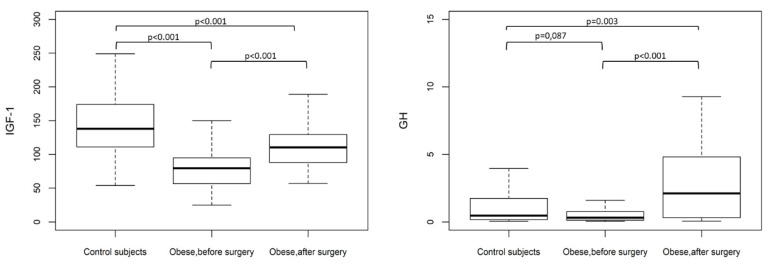
Insulin-like growth factor 1 (IGF-1) and growth hormone (GH) values (Median (IR)) in control subjects and obese patient before and twelve months after bariatric surgery.

Figure 1 shows the IGF-1 and GH values (Median (IR)) in the controls and obese patients before and twelve months after surgery. IGF-1 levels were lower in the obese group than in the controls. IGF-1 significantly increased in the obese patients twelve months after surgery-induced weight loss.

**Figure 2:**
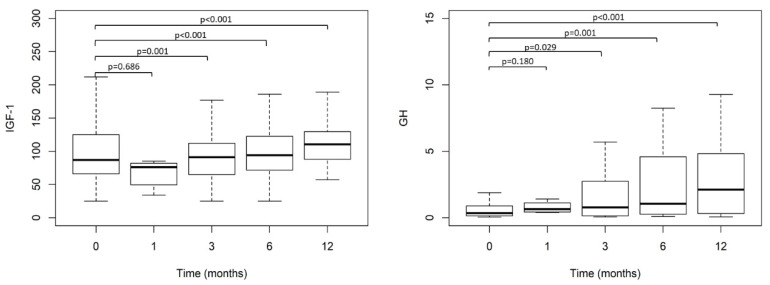
Evolution over time of IGF-1 and GH values (Median (IR)) before and after surgery (zero, one, three, six and twelve months).

Figure 2 shows the evolution over time of IGF-1 and GH (Median (IR)) before and after surgery (zero, one, three, six and twelve months) in the obese patients. The results show a progressive increase in both IGF-1 and GH values. 

**Figure 3:**
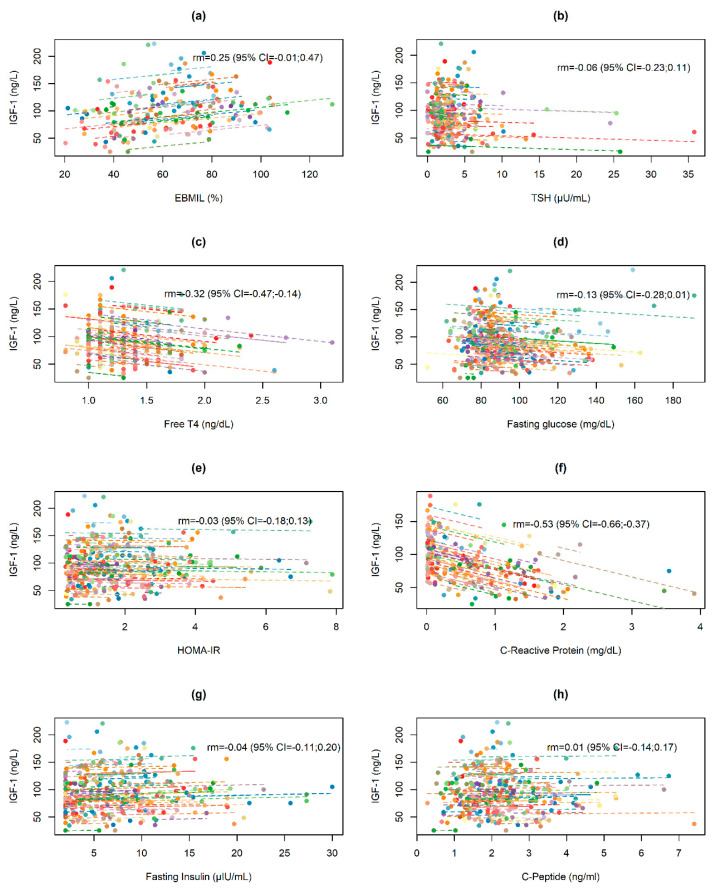
Repeated measures correlation for the overall relationship between changes in IGF-1 and (**a**) excessive BMI loss (EBMIL), (**b**) TSH, (**c**) free T4, (**d**) fasting glucose, (**e**) HOMA-IR, (**f**) C-reactive protein, (**g**) fasting insulin and (**h**) C-peptide. Obese subjects are represented by points that correspond to pre- and post-operative IGF-1 levels and the respective anthropometric, biochemical and hormonal data. Each line represents the correlation adjustment of repeated measures for each participant.

**Figure 4:**
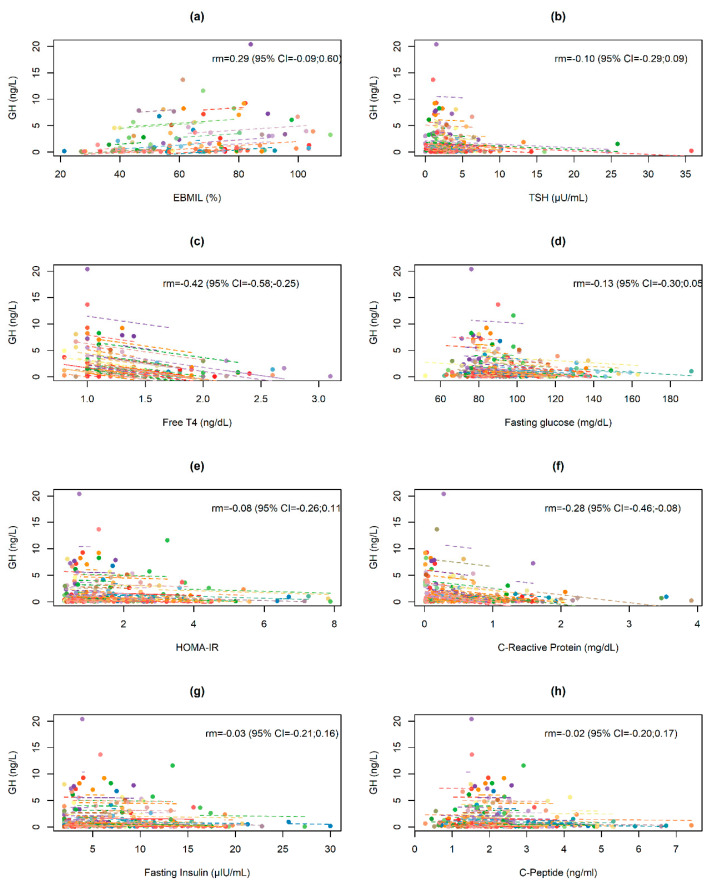
Repeated measures correlation for the overall relationship between changes in GH and (**a**) EBMIL, (**b**) TSH, (**c**) free T4, (**d**) fasting glucose, (**e**) HOMA-IR, (**f**) C-reactive protein, (**g**) fasting insulin and (**h**) C-peptide. Obese subjects are represented by points that correspond to pre- and post-operative IGF-1 levels and the respective anthropometric, biochemical and hormonal data. Each line represents the correlation adjustment of repeated measures for each participant.

Post-surgery changes in IGF-1 and GH values in relation to changes in selected anthropometric, biochemical, and hormonal data are presented in Figure 3 and Figure 4. Changes in both indices were significantly negatively correlated with changes in CRP values (rm = −0.53 and rm = −0.28, respectively) and free T4 values (within-subjects repeated measures correlation rm = −0.32 and rm = −0.42, respectively).

Bivariate GEE models confirmed the increment in both IGF-1 and GH values after surgery (linear time intercept, *p* < 0.001), with a negative association with preoperative BMI in the case of IGF-1 (B = −1.078; *p* < 0.001). 

After adjusting for preoperative BMI, free T4 and CRP in a multivariate model, only CRP was independently associated with IGF-1 values in the follow-up, with higher CRP values associated with lower IGF-1, whilst higher free T4 values were associated with lower GH values in the follow-up (Table 4).

## 4. Discussion

The main result of the present study is that IGF-1 values are decreased in patients with morbid obesity, and that weight loss after bariatric surgery drives a progressive increase in GH and IGF-1. After bariatric surgery IGF-1 and GH values were significantly negatively correlated with changes in CRP and free T4 values. After adjusting for preoperative BMI, free T4 and CRP in a multivariate model, only CRP was independently associated with IGF-1 values in the follow-up, with higher CRP values associated with lower IGF-1. Extreme obesity is a situation of functional hyposomatotropism that is partially reversed with weight loss. As far as we know, this is the first time that the GH–IGF-1 axis has been evaluated in a broad group of morbidly obese subjects before and after bariatric surgery, following its evolution over time, as patients lost weight.

In line with our results, Rasmussen et al. studied nine obese subjects after losing weight and found that considerable weight loss restores GH release profiles and serum IGF-1 [12]. Eden Engstrom studied sixty-three obese patients before and after bariatric surgery and found that GH and IGF-1 increased twelve months after weight loss [19]. In contrast with our results De Marinis et al. studied fifteen obese female patients before and after bariatric surgery-induced weight loss and found that the GH response to GH-releasing hormone markedly increased, while IGF-1 did not significantly vary [29]. Utz et al. have found that despite a linear decrease in peak stimulated GH levels with increasing weight in obese and overweight women, IGF-I levels were not proportionally reduced [18]. Moreover, weight loss in individuals with obesity after a low-energy liquid diet was associated with improvements in insulin sensitivity but IGF-1 was not significantly modified [30]. 

The mechanism of altered GH–IGF-1 axis in obesity remains unclear. Insulin has been found to reduce GH secretion in a non-human primate model [32]. In humans, low-level insulin infusions reduce the GH response to GH-releasing hormone [33]. In healthy adults, fasting insulin levels are a predictor of integrated 24-h GH concentrations [34]. Cornford et al. found that increased food intake stimulated a rapid suppression of GH secretion and the decline in GH was accompanied by an increase in plasma insulin levels [35]. In the present study we studied the correlation between the GH–IGF-1 axis and fasting insulin or the HOMA-IR index, and were unable to find any correlation. It is known that ghrelin stimulates appetite and increases circulating GH across varied patient populations [36]. Nass et al. [37] found that endogenous acylated ghrelin increases the amplitude of GH pulses. Ghrelin secretion is decreased in obesity [38] and this decrease could be responsible, at least partially, for the alteration of GH secretion observed in obese individuals [10]. The IGF-1 feedback mechanism is preserved in obesity. Administration of a low IGF-1 dose inhibits the somatotroph response to GH-releasing hormone in obese and normal subjects, indicating that somatotroph sensitivity to the inhibitory effect of IGF-1 is conserved in obesity [39]. The present results suggest that the reduced GH found in obese subjects is not due to a feedback inhibition by elevated IGF-1, since extreme obesity is associated with a decline in IGF-1 values. 

Inflammation decreases GH secretion and, in the liver, induces GH resistance due to a decrement of GH receptors and altered downstream signaling eliciting a decrease in circulating IGF-1 [40]. Obesity is a disease characterized by the presence of chronic low-grade inflammation [41]. We have found that the variation of the increased indices of inflammation such as CRP highly inversely associates with IGF-1 in obesity. In agreement with these data, a negative correlation was found between circulating IGF-1 and inflammatory index, such as fibrinogen and erythrocyte sedimentation rate in an ample group of obese subjects [42]. Moreover, in a small group of obese patients IGF-1 has been found to be negatively associated with CRP in women [43]. 

There is an important relationship between thyroid hormones, and the GH–IGF-1 axis. Thyroid hormones act at many sites from the hypothalamic control of GH release to the tissue expression of IGF-1 [44]. Circulating GH levels increased serum free triiodothyronine and decreased serum FT4 in humans [45]. GH replacement therapy in adult subjects with GH deficiency syndrome causes different alterations in thyroid function, the most common being the decrease in circulating thyroxine levels [46]. Treatment with GH in a large group of adult GH-deficient patients has been found to induce a significant reduction in FT4 [47]. The increased TSH and central resistance to thyroid hormones of obesity [7,8] could be a contributory factor to the altered GH–IGF-1 axis. A positive synchronous serum concentration correlation between TSH and GH assessed from 24-h hormone concentrations measured at 10-min intervals has been found in healthy older subjects [48]. We explored the correlation between the GH–IGF-1 axis and free T4 or TSH, and we found a significant negative correlation between GH and free T4. We believe the most plausible explanation is that the increase in GH secretion induced by weight loss could contribute to the decrement of free T4. 

The recuperation of the GH–IGF-1 axis after body weight normalization suggests an acquired and reversible disturbance; however, the reduced GH secretion in obesity may favor the retention of adipose tissue, and contribute to perpetuation of the obese state. GH’s most characteristic metabolic action is to induce adipose tissue lipolysis [49]. GH directly stimulates adipose tissue lipolysis in a GH receptor-dependent manner [50]. Clinical trials assessing the effects of GH treatment in patients with obesity have shown reductions in fat mass, especially abdominal and visceral adipose tissue depots [51]. Hormone replacement therapy with GH improves mitochondrial function, markers of cardiovascular risk and body composition, including liver fat, in abdominally obese men [52]. Furthermore, in these patients, GH administration decreases abdominal subcutaneous adipocyte size, suggesting that GH improves the health of adipose tissue [53].

GH signaling and action has been evaluated in obese patients. Hogild et al. have found that GH signaling is normal in obesity and there is increased hepatic GH sensitivity, suggesting that the blunted GH levels in obesity may protect against insulin resistance without compromising IGF-1 levels [54]. In another study Glad et al. have found that GH receptor expression was decreased in subjects with obesity compared with subjects with normal weight; GH receptor was increased in response to energy restriction and decreased in response to overfeeding. Downstream signaling genes expression were increased in subjects with obesity, decreased during energy restriction and increased during overfeeding. The authors conclude that GH signaling is perturbed in obesity [55]. The altered GH–IGF-1 axis of obesity has important clinical implications and the decreased IGF-1 values of obesity are clinically relevant. Both low and high serum IGF-1 levels are associated with increased risk of cardiovascular events in men of an advanced age [56]. Moreover, both low and high IGF-1 are associated with increased mortality in the general population [57]. Obese and overweight children and adolescents in the higher quartiles of IGF-1 had an increased risk of hypertension, hypercholesterolemia, high levels of triglycerides, and reduced HDL cholesterol. Conversely, lower IGF-1 quartiles were associated with higher blood glucose and insulin resistance [58]. These data reinforce the view that IGF-1 plays a complex role in the comorbidities of obesity [58]. It has been found that an altered somatotropic axis is associated with a worse cardio metabolic profile in obese patients [59] and that the Mediterranean diet and protein intake was associated with a better GH status [20]. Moreover, the higher IGF-1 receptor expression observed in obese children, associated with the higher IGF-1 and the lower IGFBP-1, suggest that the higher stature observed in obese children may be due to increased IGF-1 bioactivity [23]. The recovery of the deranged GH–IGF-1 axis in obesity with bariatric surgery-induced weight loss should be considered another important benefit of bariatric surgery.

We must recognize that the present study has several limitations. First, our study patients are very heterogeneous, with different comorbidities and treatments associated with obesity. Secondly, the relatively small sample size, which prevents us from subdividing it into different subgroups in order to carry out more exhaustive analysis. Thirdly, we did not consider certain aspects that could influence the study, such as the concomitant use of other medications, due again to the relatively small sample size. Fourthly, we used wide range CRP, rather than high-sensitivity CRP. However, there are several strengths to our study. We included sex- and age-matched controls to reduce the chances of misclassifying individuals due to variability in these data. We evaluated GH and IGF-1 at different time points after bariatric surgery, as most studies evaluated the variation of the GH–IGF-1 axis in fewer patients and intervals after surgery. Moreover, as far as we know, this is the first time that the GH–IGF-1 axis has been evaluated in an ample group of morbidly obese subjects before and after the evolution of surgery-induced weight loss over time. However, further studies are necessary to better understand the complex association between obesity and the GH–IGF-1 axis.

## 5. Conclusions

In conclusion, this study shows that in patients with morbid obesity, weight loss induced with bariatric surgery brings about a restoration of the altered GH–IGF-1 axis decline. This recovery of the altered GH–IGF-1 decrease is progressive over time after bariatric surgery, and significantly and independently related with C-reactive protein. Severe obesity is characterized by a functional hyposomatotropism at a central and peripheral level progressively reversible with weight loss, and low-grade chronic inflammation could be the principal mediator.

## Figures and Tables

**Table 1:** Preoperative characteristics of the obese patients and the control subjects.

	Control Subjects	Obese Subjects	*p*
	Median (IR)	Median (IR)	
**Age (years)**	42.0 (37.3; 53.0)	45.2 (39.1; 50.8)	0.794
**Sex (*n*, %)**			0.143
Female	73.2%	83.6%	
Male	26.8%	16.4%	
**BMI (kg/m^2^)**	23.2 (21.1; 24.1)	47.8 (43.8; 53.0)	<0.001
**Body fat (%)**	27.6 (20.5; 30.4)	51.1 (48.0; 54.2)	<0.001
**Type of surgery (%)**			---
Roux-en-Y gastric bypass		50.9%	
Sleeve gastrectomy		49.1%	

BMI, body mass index; IR, interquartile range.

**Table 2:** Biochemical and hormonal data in control subjects and obese patients.

	Control Subjects	Obese Subjects	*p*
	Median (IR)	Median (IR)	
**IGF-1 (µg/L)**	138.0 (111; 174)	79.5 (56.6; 95.0)	<0.001
**GH (µg/L)**	0.5 (0.2; 1.7)	0.3 (0.1; 0.8)	0.087
**TSH (µU/mL)**	1.9 (1.3; 2.5)	2.8 (1.5; 4.9)	0.002
**Free T4 (ng/dL)**	1.2 (1.1; 1.2)	1.4 (1.3; 1.7)	<0.001
**Fasting Glucose (mg/dL)**	89.0 (84.0; 95.0)	96.0 (81.5; 115.5)	0.030
**Fasting Insulin (µIU/mL)**	3.5 (2.9; 6,2)	6.0 (2.8; 9.6)	0.009
**HOMA-IR**	0.8 (0.6; 1.5)	1.4 (0.6; 2.7)	0.004

IGF-1: insulin-like growth factor 1; GH: growth hormone; TSH: thyroid-stimulating hormone; T4: thyroxine; IR, interquartile range.

**Table 3:** Anthropometric, biochemical and hormonal data in obese patients before and twelve months after bariatric surgery.

	Obese Patients Before Surgery	Obese Patients Twelve Months after Surgery	Change	*p*
	Median (IR)	Median (IR)	Median (IR)	
**IGF-1 (µg/L)**	79.5 (56.6; 95.0)	110.5 (88.0; 129.5)	−30.5 (−52.0; −11.0)	<0.001
**GH (µg/L)**	0.3 (0.1; 0.8)	2.1 (0.3; 4.8)	−2.0 (−3.6; −0.1)	<0.001
**Weight (kg)**	123.1 (105.4; 146.0)	83.5 (69.5; 98.0)	43.1 (36.7; 55.3)	<0.001
**BMI (kg/m^2^)**	47.8 (43.8; 53.0)	30.4 (26.9; 35.6)	16.5 (13.8; 19.5)	<0.001
**Body fat (%)**	51.1 (48.0; 54.2)	33.0 (25.7; 40.8)	16.7 (11.2; 20.3)	<0.001
**TSH (µU/mL)**	2.8 (1.5; 4.9)	2.3 (1.4; 3.2)	0.7 (−0.4; 2.5)	0.001
**Free T4 (ng/dL)**	1.4 (1.3; 1.7)	1.1 (1.0; 1.2)	0.3 (0.2; 0.6)	<0.001
**Fasting Glucose (mg/dL)**	96.0 (81.5; 115.5)	84.0 (77.0; 90.0)	12.0 (−2.0; 26.5)	<0.001
**Fasting Insulin (µIU/mL)**	6.0 (2.8; 9.6)	5.4 (2.9; 8.5)	0.3 (−1.8; 3.4)	0.256
**HOMA-IR**	1.4 (0.6; 2.7)	1.1 (0.6; 2.0)	0.3 (−0.4; 1.0)	0.037
**C-Peptide (ng/mL)**	2.1 (1.4; 3.2)	1.8 (1.4; 2.3)	0.1 (−0.6; 1.0)	0.194
**C-Reactive Protein (mg/dL)**	0.7 (0.4; 1.2)	0.1 (0.02; 0.3)	0.5 (0.1; 0.8)	<0.001

IGF-1: insulin-like growth factor 1; GH: growth hormone; BMI, body mass index; TSH: thyroid-stimulating hormone; T4: thyroxine; IR, interquartile range.

**Table 4:** Generalized estimating equation model examining change of IGF-1 and GH levels after bariatric surgery, adjusting for basal BMI and free T4 and C-reactive protein values in the follow-up.

	IGF-1			GH		
	B	SE	*p*	B	SE	*p*
**Intercept**	113.25	18.67	<0.001	2.06	1.06	0.052
**Linear time (months after surgery)**	1.91	0.43	<0.001	0.21	0.04	<0.001
**Basal BMI (kg/m^2^)**	−0.58	0.33	0.083	−0.01	0.02	0.679
**Free T4 (ng/dL)**	3.15	5.34	0.555	−0.62	0.31	0.045
**C-Reactive Protein (mg/dL)**	−12.29	3.23	<0.001	0.01	0.20	0.997

B: unstandardized beta; SE: standard error; GH: growth Hormone; IGF-1: insulin-like growth factor 1; BMI: body mass index; T4: thyroxine.
